# Semantic Segmentation of a Printed Circuit Board for Component Recognition Based on Depth Images

**DOI:** 10.3390/s20185318

**Published:** 2020-09-17

**Authors:** Dongnian Li, Changming Li, Chengjun Chen, Zhengxu Zhao

**Affiliations:** School of Mechanical & Automotive Engineering, Qingdao University of Technology, Qingdao 266525, China; dongnianli@qut.edu.cn (D.L.); changmingli168@163.com (C.L.); zhaozhengxu@qut.edu.cn (Z.Z.)

**Keywords:** PCB, component recognition, semantic segmentation, pixel classification, random decision forest, depth image

## Abstract

Locating and identifying the components mounted on a printed circuit board (PCB) based on machine vision is an important and challenging problem for automated PCB inspection and automated PCB recycling. In this paper, we propose a PCB semantic segmentation method based on depth images that segments and recognizes components in the PCB through pixel classification. The image training set for the PCB was automatically synthesized with graphic rendering. Based on a series of concentric circles centered at the given depth pixel, we extracted the depth difference features from the depth images in the training set to train a random forest pixel classifier. By using the constructed random forest pixel classifier, we performed semantic segmentation for the PCB to segment and recognize components in the PCB through pixel classification. Experiments on both synthetic and real test sets were conducted to verify the effectiveness of the proposed method. The experimental results demonstrate that our method can segment and recognize most of the components from a real depth image of the PCB. Our method is immune to illumination changes and can be implemented in parallel on a GPU.

## 1. Introduction

The printed circuit board (PCB) is an important part of modern electronic products. The quality of the PCB determines the quality of the product. In particular, the quality of the assembly of the components on the PCB directly influences the performance and service life of electronic equipment. Traditional manual inspection of PCBs is inefficient, and has a high error rate, which makes it difficult to adapt to industrial situations. Automatic inspection based on machine vision is increasingly applied in fields related to PCB inspection. Thus, automatic visual inspection of PCBs has become a popular research topic in the area of industrial inspection. In addition, the flexibility of modern automatic production systems could be improved significantly if the production equipment was able to effectively and automatically recognize and inspect the PCB.

In recent years, many researchers have carried out studies on PCB inspection based on machine vision. Among them, a lot of studies [1,2,3,4,5,6,7,8,9,10,11] have focused on the inspection of solder joints, which is both important and challenging. Researchers have proposed a number of methods for solder joint inspection that use neural networks [1,2], fuzzy rules [3], Boolean rules [4], deep learning [5], support vector machines [6], decision trees [7], principle component analysis [8], modal analysis [9], etc. Component placement inspection is another significant and challenging problem, and it is the basis of other PCB inspections, such as the inspection of solder joints. Many defects are caused by errors in PCB component placement, such as missing or misaligned components, or the incorrect rotation of the components. In addition, the techniques and methods derived from the research on component placement inspection can also be used in automated PCB recycling. With regard to the inspection of component placement, research needs to be conducted on segmentation, detection, and recognition of the components in the PCB to locate and identify the components. Many studies [12,13,14,15,16,17,18,19,20,21,22,23,24] have been carried out to solve this problem. Crispin et al. [12] proposed a genetic algorithm template-matching method to locate and identify PCB components from images to the component placement. They used grey-model templates for PCB components. Zeng et al. [13] proposed a method to locate PCB components according to the color distribution of solder joints. By using three layers of ring-shaped LEDs as illumination, they recognized and located the solder joints based on their color distribution patterns. Then, they located each component by clustering all the solder joints of the component. The method uses PCB color images as input. Herchenbach et al. [14] proposed a framework for segmentation and classification of through-hole components on PCBs to enable the automatic recycling of PCBs by using both RGB (Red Green Blue) and depth frames from the Microsoft Kinect sensor as input. They used a multi-threshold approach to create segmentation hypotheses of components and searched for the best hypothesis using an optimization step. However, they misclassified a great number of PCB segments as components. Li et al. [15] divided the surface mounted devices (SMDs) mounted on PCBs into two groups: small devices and integrated circuits (ICs). They used assembly print-based segmentation for small devices and color distribution-based segmentation for ICs, respectively. Lim et al. [16] used a convolution neural network (CNN) for classification of SMDs mounted on PCBs. They extracted the device regions from device images for classification. However, the segmentation of device images from PCB images was not presented in [16].

In this paper, we propose a PCB semantic segmentation method based on depth images, to segment and recognize components in the PCB through pixel classification. Machine vision based on depth images has high robustness since it is not influenced by the illumination and the texture of the target object [25]. Pixel classification methods based on depth images have been applied in the field of human body pose recognition [26,27,28] and hand gesture recognition [29,30,31,32]. Shotton et al. [26,27] constructed a large synthetic depth image database with automatic pixel labeling by using motion capture data of human bodies. They used the database to train a random forest and estimated the approximate poses of different parts of the human body through pixel classification based on depth images. Keskin et al. [29,30] estimated hand gestures from depth images by using a pixel classification method with random forests.

In this paper, a random forest pixel classifier based on depth images is applied to the semantic segmentation of a PCB, which segments and recognizes the components in the PCB through pixel classification. We first set up an image training set and an image test set for the PCB with graphic rendering. Then, depth difference features were extracted from depth images in the training set, and the random forest pixel classifier was trained by the depth difference features, thus establishing a mapping relationship from depth difference features to pixel classification labels. Finally, by using the constructed random forest pixel classifier, we carried out semantic segmentation for the PCB to segment and recognize components in the PCB through pixel classification. The proposed method can be further used to inspect the placement of the components in the PCB. In addition, the proposed method can be used in automated PCB recycling [14,15,33].

The main contributions of this paper are as follows:

- A method for segmenting and recognizing components on a PCB is proposed by using a random forest pixel classifier based on depth images.
- A technique is developed to synthesize the PCB training image set automatically based on the graphic rendering engine OpenSceneGraph (OSG).
- A mechanism for extracting depth difference features is constructed by using a series of concentric circles centered at the depth pixel to be predicted.

This paper is organized as follows: Section 2 outlines the framework of the proposed method. Section 3 describes the construction of the image training set and test set. Section 4 describes the extraction of depth difference features. The training and predicting process of the random forest pixel classifier is presented in Section 5. The experimental results and discussions are provided in Section 6. Section 7 concludes the paper and presents the future work.

## 2. The Framework of the Method

**Figure 1:**
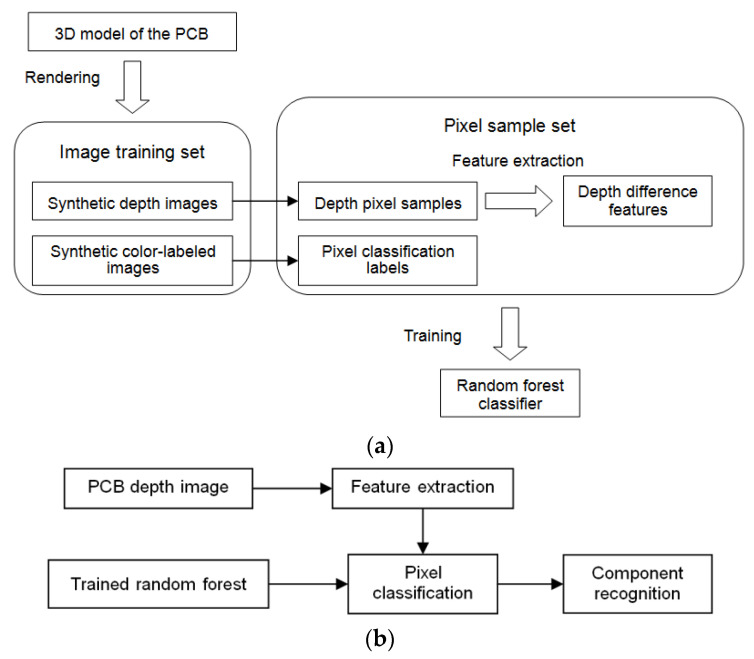
The framework of the proposed method. (**a**) Training. (**b**) Testing.

In this paper, semantic segmentation of the PCB was performed with a pixel classification method using random forests to achieve segmentation and recognition of the components on the PCB. The technological framework of the method is shown in Figure 1.

First, we set up an image training set and an image test set for the PCB, which covered the depth image samples and the corresponding color-labeled image samples. The image training set was synthesized by computer graphic rendering based on a prebuilt 3D model of the PCB. The image test set includes both synthetic image samples and real image samples. Pixels in the training images were randomly selected as pixel training samples. The depth difference features of the selected pixels were extracted from the depth images in the training set, and the classification labels of the pixels were extracted from the corresponding color-labeled images.

Then, a random forest classifier was trained by the extracted depth difference features, which established a mapping relationship from the depth difference features to the pixel classification labels. The trained random forest classifier was tested by predicting the depth images in the test set, and the optimal configuration of parameters for the random forest were acquired by repeated training and testing. Finally, semantic segmentation of the PCB was performed by the established random forest pixel classifier to segment and recognize the components in the PCB. Thus, the placement of the components in the PCB could be inspected.

## 3. Construction of the Image Training Set and Test Set

**Figure 2:**
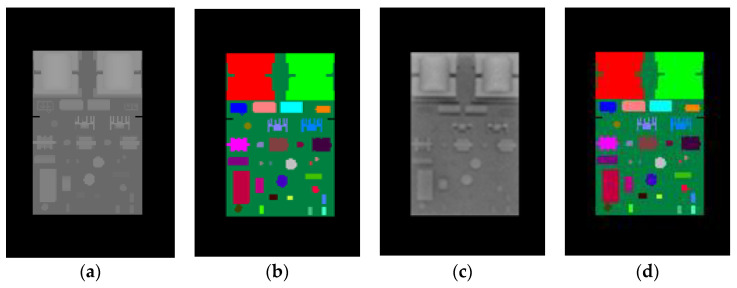
Depth images and corresponding color-labeled images of the printed circuit board (PCB). (**a**) Synthetic depth images. (**b**) Synthetic color-labeled images. (**c**) Real depth images. (**d**) Real images labeled with colors.

In this paper, 3D computer graphic rendering was used to synthesize the image sample set required to train the random forest classifier. First, we constructed a 3D model of the PCB by using a CAD modeling software, which was then put into the visualization modeling software MultiGen-Paradigm Creator through the .obj middle file format. In the Creator, components denoted $\left\{P_{i}|i=1,\ldots ,N\right\}$ of the PCB were labeled in different colors. Each color corresponds to one classification label $l_{i}$. N is the number of components in the PCB. Next, the PCB model with color labels was loaded into the open-source graphic rendering engine, OSG. The pose states of the PCB were sampled within a certain range in the pose space that the PCB lies in, thus obtaining the pose sample set $\left\{s_{j}|j=1,\ldots ,M\right\}$. *M* is the number of pose samples of the PCB. For each pose sample $s_{j}$, a depth image (Figure 2a) and its corresponding color-labeled image (Figure 2b) of the PCB under this pose were synthesized with OSG off-screen rendering through a frame buffer object (FBO). In this way, we constructed a depth image sample set and a corresponding color-labeled image sample set, which are required to train the random forest classifier.

The synthetic depth images were generated by data stored in the GPU depth buffer, the Z-buffer, while the synthetic color-labeled images were generated by data stored in the GPU color buffer. The image test set for the random forest included both synthetic image samples and real image samples. Like the training images, the synthetic test images were also generated through computer graphic rendering. To collect the real images for testing, we acquired the depth images of the real PCB by a Kinect 2.0 depth sensor (Figure 2c) and manually obtained the corresponding color-labeled images by using an image processing software to label the acquired depth images with colors (Figure 2d).

## 4. Extraction of Depth Features

During feature extraction, a certain number of pixels were selected randomly from each depth image in the image training set to generate the pixel sample set. Depth difference features of the pixel samples were used to train the random forest pixel classifier. Given a depth pixel ***x***, the depth difference feature $f_{\theta }$ is defined as

(1) $$f_{\theta }=d\left(x+\frac{u_{1}}{d(x)}\right)-d\left(x+\frac{u_{2}}{d(x)}\right)$$

where the parameters $\theta =(u_{1},u_{2})$ describe two 2D pixel offset vectors ($u_{1}$ and $u_{2}$) relative to the depth pixel ***x***, and d(x) is the corresponding depth value of depth pixel ***x*** in the depth image. Two offset vectors $u_{1}$ and $u_{2}$ are normalized by dividing with d(x), ensuring depth invariance of the feature value, $f_{\theta }$. In other words, $f_{\theta }$ is not influenced by the distance from the object to the camera. If the offset pixel lies in the background region or beyond the image boundaries, the corresponding depth value is set to C+d(x), where C is a large constant and d(x) is the depth value of the original depth pixel ***x***. These depth difference features only provide weak classification signals, but the combination of these features with a random forest classifier will enable the recognition of different parts in an object.

**Figure 3:**
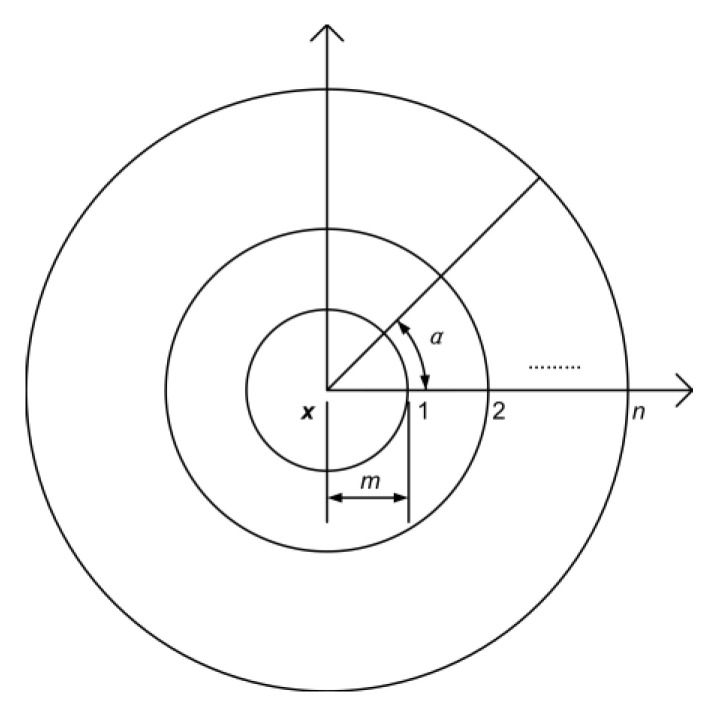
The concentric circles for depth feature extraction.

In this paper, as shown in Figure 3, a set of concentric circles centered at depth pixel ***x*** were set up for depth feature extraction. A set of offset vectors were selected on each concentric circle, according to Equations (2) and (3).

(2) $$A=\left\{\alpha |\alpha =\frac{2i\pi }{N},i=1,2,\ldots ,N,N=8n\right\}$$

(3) U={u|u=M(cosα,sinα),α∈A,M=mn}

where ***A*** is a set of *N* equal angles on the *n*th concentric circle, and ***U*** is a set of offset vectors that are selected on the *n*th concentric circle. M=mn is the modulus of the offset vector ***u***, which is also the radius of the *n*th concentric circle and *m* is a constant basis of modulus *M*. In this paper, eight concentric circles were set up, so n=1,2,…,8. A total of 288 offset vectors were selected according to Equations (2) and (3). The extracted depth difference features include unitary features and binary features. When extracting the unitary depth difference features, we let $u_{2}=0$ and calculated the depth difference between each offset point and the depth pixel ***x***. The 288 offset vectors on the eight concentric circles provided 288 unitary features. When the binary depth difference features were extracted, 1800 pairs of offset vectors were chosen randomly from the 288 offset vectors, and the depth difference between two offset points was calculated. Therefore, a total of 2088 depth difference features were extracted for each depth pixel ***x*** for classification.

## 5. Random Decision Forest

**Figure 4:**
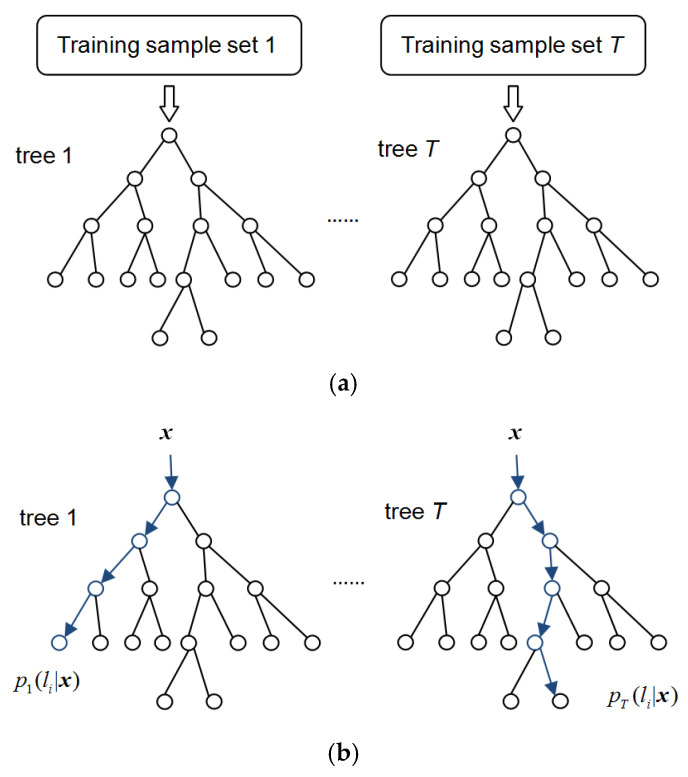
The training and predicting process of the random decision forest. (**a**) The training process. (**b**) The predicting process.

In this paper, semantic segmentation of the PCB was performed by using a random forest pixel classifier to segment and recognize components on the PCB. The random forest classifier is trained by depth difference features extracted from synthetic depth images. Random decision forest [34,35] is a fast and effective multiclass classifier that comprises multiple decision trees. As shown in Figure 4, each decision tree is composed of many branch nodes and leaf nodes. Each branch node corresponds to a feature $f_{\theta }$ and contains a threshold τ. Each leaf node contains a probability distribution of classification label $l_{i}$. When pixel ***x*** is predicted by the *t*th decision tree for classification, the depth difference feature of pixel ***x*** is compared against the corresponding threshold at each branch node of decision tree *t*. Based on the results of the comparison, pixel ***x*** is classified to the left or right sub-node and finally assigned to a leaf node. The probability distribution $p_{t}(l_{i}|x)$ corresponding to this leaf node makes a prediction for pixel ***x***. The probability distributions gained by all the decision trees in the forest are averaged at the end to make the final decision for pixel ***x*** according to Equation (4).

(4) $$p(l_{i}|x)=\frac{1}{T}\sum_{t=1}^{T}p_{t}(l_{i}|x)$$

A random forest trains and predicts samples by several random decision trees, which can effectively avoid the over-fitting problem that happens frequently for single decision tree. The training samples used in each tree are sampled randomly with replacement from the general training sample set. The features used for the training of each branch node in the decision trees are sampled randomly with a certain proportion from all features without replacement. In this paper, the optimal parameters for random forest were determined by repeated training and testing. The parameters of concern here mainly include the maximum depth of the decision trees and the number of decision trees. The training and predicting processes of the random forest classifier are summarized as follows:

**Training:**

For tree t=1−T:

1. The training samples are selected randomly with replacement from the general training set, constructing a training sample set for the current decision tree. Start training from the root node.
2. For the current branch node, *n* features $\left\{f_{\theta }\right\}$ are selected randomly from all features without replacement. For each selected feature $f_{\theta }$, samples in the sample set *S* of the current node are divided into the left subset $S_{L}$ and the right subset $S_{R}$, according to the comparison between $f_{\theta }$ and the corresponding threshold τ, as shown in Equation (5). $S_{L}$ and $S_{R}$ are allocated to the left subnode and right subnode of the current node, respectively.
  (5) $$\{\begin{array}{c} S_{L}(\theta ,\tau )=\left\{x|f_{\theta }(x)<\tau \right\}\\ S_{R}(\theta ,\tau )=\left\{x|f_{\theta }(x)\geq \tau \right\} \end{array}$$
  From the *n* selected features, search for the feature $f_{\theta }$ and threshold τ that split the samples best according to Equations (6) and (7).
  (6) $$Gini(S)=1-\sum_{i=1}^{N}p_{i}^{2}$$
  (7) $${\left(\theta ,\tau \right)}^{*}=\underset{\theta ,\tau }{\mathrm{argmax}}(Gini(S)-\frac{\left|S_{L}\right|}{\left|S\right|}Gini(S_{L})-\frac{\left|S_{R}\right|}{\left|S\right|}Gini(S_{R}))$$
  where Gini(S) is the Gini coefficient of sample set *S*, and $p_{i}$ is the proportion of class *i* samples in the sample set.
3. If the current node fits the termination criterion, it is set as a leaf node. This leaf node stores a probability distribution $p(l_{i})$ of classification label $l_{i}$, which calculated by the proportion of $l_{i}$ samples in the current leaf node.
4. If some nodes have not been split or set as a leaf node, turn to Step 2.

**Prediction:**

For tree t=1−T:

1. Start from the root node of the current decision tree, classify sample ***x*** into the left subnode or right subnode of the current node according to the comparison between the corresponding feature $f_{\theta }$ and its threshold τ.
2. Repeat Step 1 until arriving at a leaf node of the decision tree. Output the corresponding probability distribution $p_{t}(l_{i}|x)$ to make the prediction of sample ***x*** for the current decision tree.

Finally, the probability distributions gained by all *T* trees are averaged, and the class label with the maximum probability is made as the output of the random forest.

## 6. Experiments and Discussions

**Figure 5:**
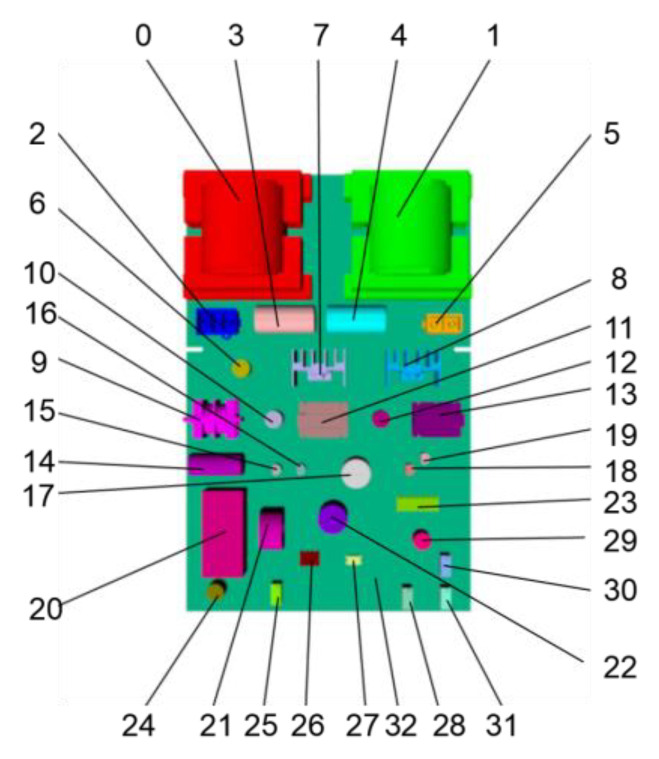
Color labels of the PCB components.

The effectiveness of the proposed method was verified by semantic segmentation and component recognition of a typical PCB, an ultrasonic power board. A 3D model of the PCB was constructed with SolidWorks and then loaded into Multigen-Paradigm Creator to label its components with different colors. Then, an image training set for random forest training and the synthetic images for test were generated through the rendering of the PCB model with OSG. The real depth images of the PCB were acquired by a Kinect 2.0 depth sensor. The color-labeling of the acquired real depth images was achieved manually with an image processing software. The color labels of the components on the PCB are shown in Figure 5. The training program of the random forest pixel classifier was developed on the Linux platform by using the g++ compiler and OpenCV. The classifier was trained on a workstation with two Intel Xeon 10-core E5-2630 v4 2.2GHz CPUs, 64GB memory, and two Nvidia Titan X GPUs.

### 6.1. Selection of Training Parameters

**Figure 6:**
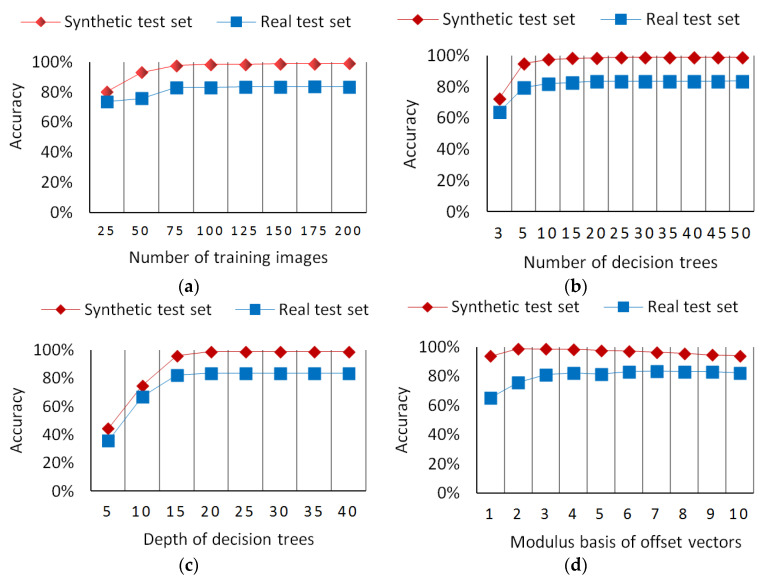
Training parameters vs. pixel classification accuracy. (**a**) Number of training images. (**b**) Number of decision trees. (**c**) Depth of decision trees. (**d**) Modulus basis of offset vectors.

To select the optimal training parameters for the random forest classifier, we designed the following four groups of experiments. In these experiments, the training samples were totally selected from the synthetic image set. A total of 2000 pixel samples were randomly selected from each depth image in the image training set. The image test set was divided into a synthetic test subset and a real test subset. The synthetic test set is composed of 40 depth images that were randomly selected from the synthetic image set. The real test set is composed of 10 images that were randomly selected from the real image set. The experimental results are shown in Figure 6.

#### 6.1.1. Number of Training Images

A certain number of training images provide the basis by which the classifier obtains recognition capabilities. However, the more training images, the greater the required memory and training time, thus it is necessary to determine the optimal number of training images by experimenting with the random forest classifier. In these experiments, the number of decision trees and the depth of each decision tree were both set to 30. The number of training images was increased from 25 to 200 gradually in increments of 25 for each experiment. The experimental results are shown in Figure 6a. In the beginning, the pixel classification accuracy increases quickly with the increase in the number of training images. After 75 training images, the pixel classification accuracy of the trained random forest becomes stable.

#### 6.1.2. Number of Decision Trees

The random decision forest has multiple random decision trees, which makes it capable of solving the problem of overfitting. The classification result of the random forest is determined by votes from all random decision trees. Thus, the number of decision trees can influence the performance of the random forest. If there are only a few decision trees, the random forest classifier is easily susceptible to overfitting. However, the more decision trees, the greater the training time and training cost. In this paper, we designed two groups of experiments on synthetic and real test images to determine the optimal number of decision trees. In these experiments, a total of 200 synthetic depth images were used for training, and the depth of the decision trees was set to 30. The number of decision trees was increased gradually from 3 to 50. The experimental results are shown in Figure 6b. With the increase in the number of decision trees, the pixel classification accuracy of the trained random forest increases quickly in the beginning and then becomes stable after 10 decision trees.

#### 6.1.3. Depth of Decision Trees

The depth of the decision trees can significantly influence the pixel classification accuracy of a random forest. Therefore, it is necessary to determine the optimal depth of the decision trees to achieve the best classification accuracy. Two groups of experiments on synthetic and real test images were designed to determine the optimal depth of decision trees. In these experiments, a total of 200 synthetic depth images are used for training, and the number of decision trees was set to 30. The depth of the decision trees was increased gradually from 5 to 40 in increments of 5 for each experiment. The experimental results for the depth of the decision trees are shown in Figure 6c. With the increase in the depth of the decision trees, the pixel classification accuracy of the trained random forest increases quickly in the beginning and becomes stable after the depth reaches 20.

#### 6.1.4. Modulus Basis of Offset Vectors

The modulus values of the offset vectors reflect the scale of the offset vectors. Therefore, it is necessary to determine the optimal values of each offset vector modulus through experiments. The offset vector moduli M=mn, which are also the radii of the concentric circles used for extracting depth difference features, are integral multiples of the modulus basis *m*. Thus, we only designed experiments to determine the optimal value of the modulus basis *m*. Two groups of experiments were conducted in this study to test the influences of *m* on the pixel classification accuracy of the synthetic test set and the real test set. In these experiments, a total of 200 synthetic depth images were used for training. The number of decision trees and the depth of each decision tree were both set to 30. The value of *m* was increased from 1 to 10 in increments of 1 for each experiment. The experimental results are shown in Figure 6d. It can be seen from the results that the value of *m* influences the pixel classification accuracy of the synthetic test set slightly, but it could significantly influence the pixel classification accuracy of the real test set. For the synthetic test set, with an increase in *m*, the pixel classification accuracy of the random forest first increases and then decreases, and reaches a peak when *m* is set to 2. However, for the real test set, the pixel classification accuracy of the random forest obtains its highest value when *m* is set to 7.

### 6.2. PCB Semantic Segmentation and Component Recognition

**Figure 7:**
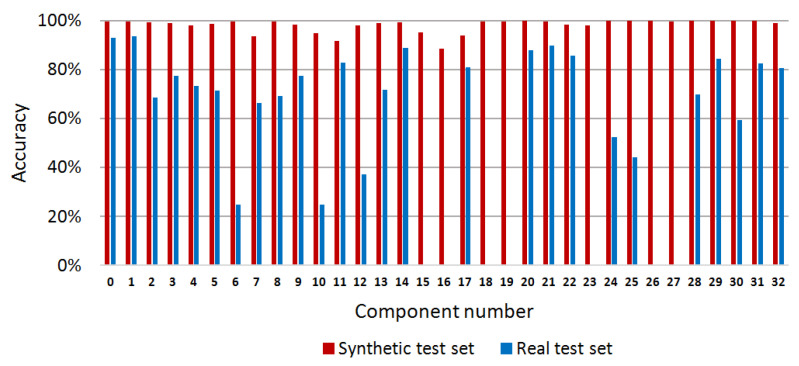
Pixel classification accuracy of the random forest classifier for each PCB component on synthetic and real data.

In this paper, semantic segmentation of the PCB was performed through pixel classification by a random forest to segment and recognize each component in the PCB. To verify the effectiveness of the proposed method, we designed two experiments on synthetic and real test images. Specifically, 40 depth images and the corresponding label images were selected randomly from the synthetic image set as the synthetic test set. Ten depth images and the corresponding label images were selected randomly as the real test set from the real image set. In these experiments, a total of 200 synthetic depth images were used for training. For experiments on the synthetic test set, we constructed the image training set with the yaw angles of the PCB model sampled uniformly in the range of 360° and the pitch angles were sampled uniformly in the range of (−30°,30°). However, for experiments on the real test set, to counter the effects of the image noises on the accuracy of our method, we narrowed down the variety of the sampled training images and the yaw angles and the pitch angles of the PCB model were both sampled uniformly in the range of (−15°,15°). In all of the experiments, the number of decision trees and the depth of each decision tree were both set to 30. For the synthetic test set, the modulus basis *m* of the offset vectors was set to 2, whereas for the real test set, *m* was set to 7. The test program runs on a laptop with a 4-core Core i5 2.9GHz CPU, 8.0GB memory, and a Nvidia GTX 950M GPU. Our method takes about 0.9s on average to process one PCB image, which involves the time for feature extraction and the time for the predicting process of the random forest. The average accuracy of the pixel classification for the proposed method is 98.96% on the synthetic test images and 83.64% on the real test images. The pixel classification accuracy of each component is shown in Figure 7, which is calculated according to Equation (8).

(8) $${\mathrm{AC}}_{i}=\frac{TP_{i}}{TP_{i}+FP_{i}}$$

where ${\mathrm{AC}}_{i}$ is the pixel classification accuracy of component *i*, $TP_{i}$ is the number of true-positive pixel classification results for component *i* and $FP_{i}$ is the number of false-positive pixel classification results for component *i*.

**Figure 8:**
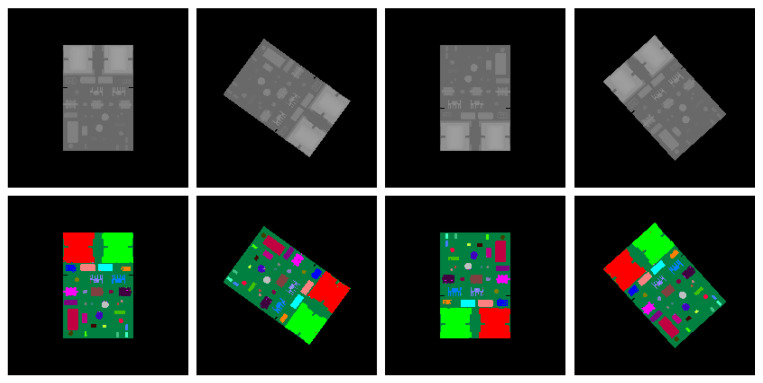
Synthetic depth images and the corresponding pixel classification images. The first row: synthetic depth images for test. The second row: the corresponding pixel classification images generated by the trained random forest pixel classifier.

**Figure 9:**
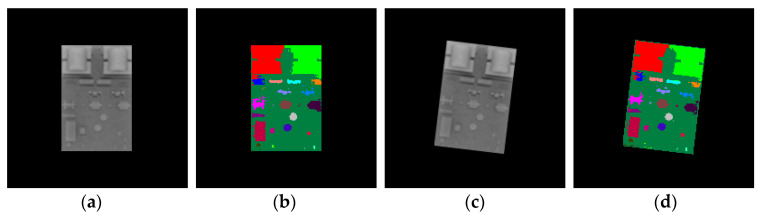
Real depth images and the corresponding pixel classification images. (**a**,**c**) Real depth images for test. (**b**,**d**) The corresponding pixel classification images generated by the trained random forest pixel classifier.

Sample results of the synthetic test set are shown in Figure 8 whereas the results of the real test images are shown in Figure 9. It can be seen from Figure 8 that for synthetic depth images all of the components on the PCB can be accurately segmented and recognized using the proposed method. For real depth images of the PCB, most of the components can also be segmented and recognized using the proposed method, as shown in Figure 9. However, Kinect 2.0 is a low-cost consumer-level electronic sensor. Due to the limited precision of the Kinect 2.0 depth sensor, several small, thinner components, such as component 6, 15, 16 18, 19, 26, and 27, cannot be correctly detected from the real depth image. Future research will apply a high-precision industrial depth sensor to capture real depth images.

**Figure 10:**
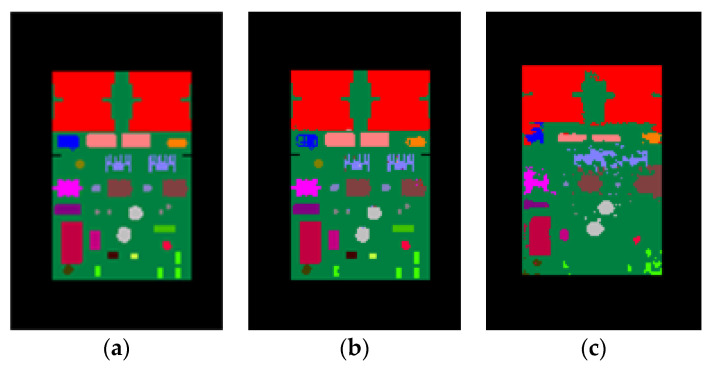
Component type labels used as the output labels. (**a**) The ground truth color-labeled image. (**b**) The results for the synthetic depth image. (**c**) The results for the real depth image.

The goal of our system is to inspect the placement of the components mounted on the PCB. We need to ensure that the correct components are mounted in the right positions. Thus, the output labels of the PCB do not need to be the component instance labels. So, we labeled the same type of components (component 0 and 1, 3 and 4, 7 and 8, etc.) rather than the component instance with a same color, and conducted some tests. Figure 10 shows our results for synthetic and real images with component type labels used as the output labels. The average accuracy of pixel classification was 99.26% on the synthetic test images and 83.84% on the real test images, slightly higher than their counterparts when using component instance labels as the output labels. It can be seen that the results in Figure 10 are nearly equal in quality to the results in Figure 8 and Figure 9 where component instance labels are used as the output labels. In this paper, we used the depth difference features that were extracted based on a set of concentric circles for training and predicting. These features make use of the information from the neighboring depth pixels to predict the given depth pixel and to decide which component instance or component type the given depth pixel belongs to. Component type segmentation fits better with the goal of our system, whereas component instance segmentation allows us to analyze the accuracy of each specific component instance.

### 6.3. Tests with Missing or Misplaced Components

**Figure 11:**
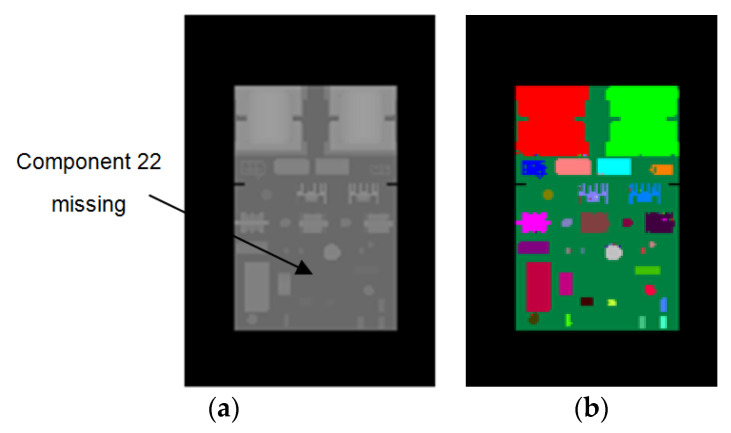
PCB with component 22 missing. (**a**) Synthetic depth image. (**b**) The resulting pixel classification image of (**a**).

**Figure 12:**
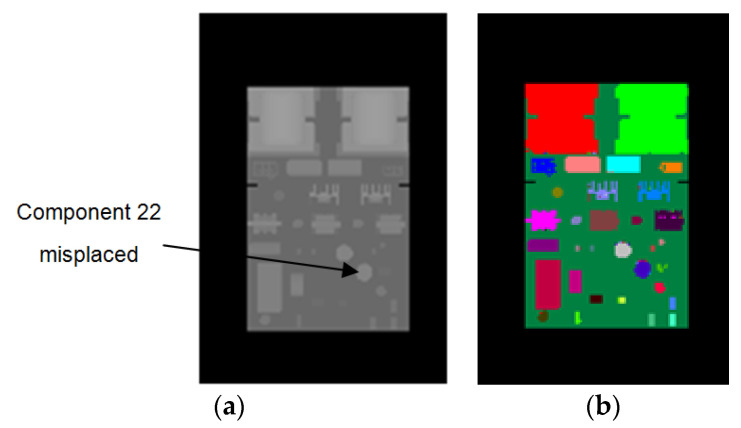
PCB with component 22 misplaced. (**a**) Synthetic depth image. (**b**) The resulting pixel classification image of (**a**).

**Figure 13:**
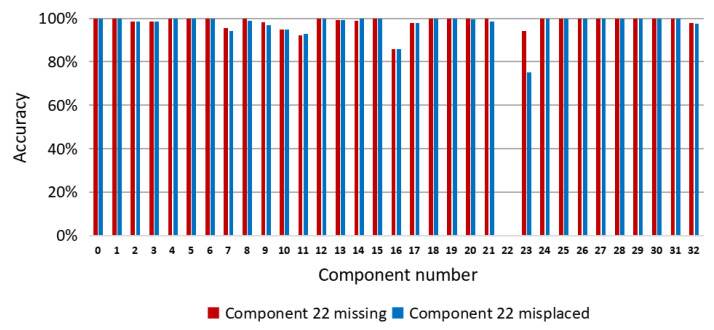
Pixel classification accuracy of each component on PCBs with component 22 missing and misplaced, which is calculated based on the color-labeled image of the correctly mounted PCB.

To evaluate the performance of our method for component placement inspection, we conducted experiments on PCBs with missing or misplaced components. We used the synthetic images of the correctly mounted PCB to train the random forest pixel classifier. During the inspection, we used the trained classifier to predict the depth images of the PCBs with missing or misplaced components. Then, we compared the resulting pixel classification images of these PCBs with the color-labeled image of the correctly mounted PCB (as shown in Figure 2b) and calculated the accuracy of each component according to Equation (8). By analyzing the accuracies of the components, the missing or misplaced components could be detected. Since the real images acquired by the Kinect 2.0 depth sensor have limited depth precision, we used synthetic depth images for testing in these experiments. Figure 11 shows a PCB with component 22 misplaced, whereas Figure 12 shows a PCB with component 22 missing. Figure 13 shows the accuracy of each component on these PCBs calculated based on the color-labeled image of the correctly mounted PCB. It can be seen from Figure 13 that the accuracy of component 22 is abnormal in both situations. In this way, component 22 can be identified as the missing or misplaced component.

### 6.4. Tests with Other PCBs

To further verify the effectiveness of our method, we also conducted experiments on other PCBs: a television motherboard and a controller board. Since the real images acquired by the Kinect 2.0 depth sensor have a limited depth precision, the synthetic depth images were used for testing in these experiments. In these experiments, 200 synthetic depth images were used for training. The number of decision trees and the depth of each decision tree were both set to 30. The modulus basis *m* of the offset vectors was set to 2.

**Figure 14:**
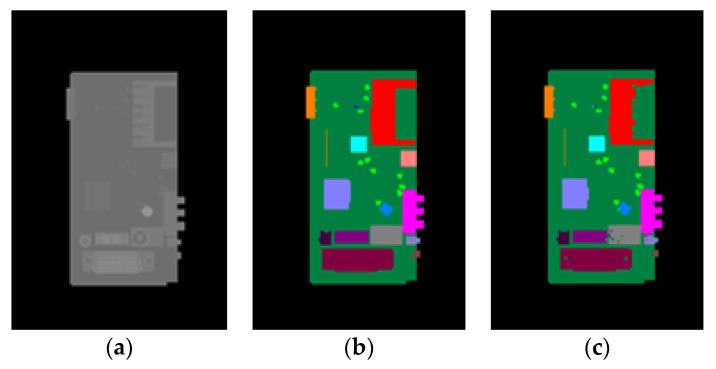
The results of our method for a television motherboard. (**a**) Synthetic depth image. (**b**) Color-labeled image. (**c**) The resulting pixel classification image of (**a**).

**Figure 15:**
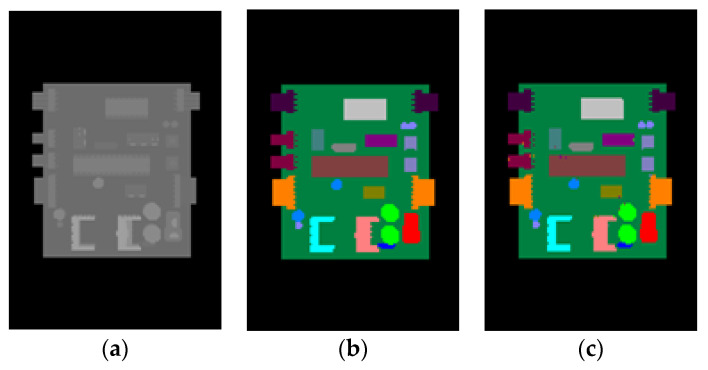
The results of our method for a controller board. (**a**) Synthetic depth image. (**b**) Color-labeled image. (**c**) The resulting pixel classification image of (**a**).

The average accuracies of pixel classification for our method for these two boards were both above 98%. In Figure 14 and Figure 15, a synthetic depth image for testing and the corresponding pixel classification image are shown for these two boards, respectively. It can be seen from Figure 14 and Figure 15 that our method was also effective for the semantic segmentation of other PCBs.

## 7. Conclusions

This paper proposes a PCB semantic segmentation method based on depth images to segment and recognize the components on the PCB. An image training set and image test set of the PCB were constructed. Depth difference features were extracted from the synthetic depth images in the training set to train a random forest classifier. The trained random forest pixel classifier was applied for semantic segmentation of the PCB. Components in the PCB were segmented and recognized through pixel classification. According to experimental results for the synthetic and real test sets, the proposed method can achieve effective segmentation and recognition of PCB components. The average pixel classification accuracy of the proposed method achieves 98% for synthetic depth images and 83% for real depth images. The proposed method can be used to inspect the placement of the components in the PCB. In addition, the proposed method can also be used in automated PCB recycling. During the recycling of the PCBs, the reusable components and precious materials need to be recovered and the toxic substances should be separated. The segmentation of the components mounted on a PCB is a first step for automated PCB recycling that is based on a comprehensive analysis of the PCBs.

The proposed method can segment and recognize most of the components from a real depth image of the PCB. However, due to the limited precision of the Kinect 2.0 depth sensor, some small, thinner components may not be correctly detected from the real depth image. Kinect 2.0 is a low-cost, consumer-level electronic sensor. In future research, we will change to a high-precision industrial depth sensor for capturing real depth images. In this paper, the pixel classifier is trained by synthetic data. The image training set is synthesized automatically by computer graphic rendering based on the 3D model of the PCB, but color labeling of the 3D model of the PCB and its components requires manual work. In future research, we plan to achieve automatic color labeling through secondary development of the 3D CAD software systems. On this basis, the image training set could be generated completely automatically in industrial production. The proposed method is not a real-time method for prediction. However, the feature extraction and random forest can both be implemented in parallel on a GPU. Future research will accelerate the system by GPU programming.

## Symbols

$l_{i}$	Classification label for component *i*
$s_{j}$	Pose sample *j* of the PCB
x	Pixel coordinates of the given depth pixel to be predicted
u	Pixel offset vectors relative to depth pixel ***x***
d(x)	The corresponding depth value of depth pixel ***x***
θ	A pair of pixel offset vectors ($u_{1}$ and $u_{2}$) for depth feature extraction
$f_{\theta }$	Depth difference feature at parameter θ
τ	The threshold corresponding to $f_{\theta }$ at the branch node
C	A large constant
α	The angle of the offset vector
n	Concentric circle number
m	A constant basis for offset vector moduli
A	A set of *N* equal angles on the *n*th concentric circle for depth feature extraction
U	A set of offset vectors that are selected on the *n*th concentric circle
t	Decision tree number
$S_{L}$	The left sample subset of the current branch node
$S_{R}$	The right sample subset of the current branch node
${\mathrm{AC}}_{i}$	The pixel classification accuracy of component *i*
$TP_{i}$	The number of true-positive pixel classification results for component *i*
$FP_{i}$	The number of false-positive pixel classification results for component *i*
